# Multiscale selection in spatially structured populations

**DOI:** 10.1098/rspb.2023.2559

**Published:** 2024-05-29

**Authors:** Hilje M. Doekes, Rutger Hermsen

**Affiliations:** ^1^ Theoretical Biology Group, Department of Biology, Utrecht University, Padualaan 8, 3584 CH Utrecht, The Netherlands; ^2^ Laboratory of Genetics, Department of Plant Sciences, Wageningen University, Droevendaalsesteeg 1, 6708 PB Wageningen, The Netherlands; ^3^ Centre for Complex Systems Studies, Utrecht University, Leuvenlaan 4, 3584 CE Utrecht, The Netherlands

**Keywords:** spatial structure, evolution, self-organization, Price’s equation, altruism, pathogen transmissibility

## Abstract

The spatial structure of populations is key to many (eco-)evolutionary processes. In such cases, the strength and sign of selection on a trait may depend on the spatial scale considered. An example is the evolution of altruism: selection in local environments often favours cheaters over altruists, but this can be outweighed by selection at larger scales, favouring clusters of altruists over clusters of cheaters. For populations subdivided into distinct groups, this effect is described formally by multilevel selection theory. However, many populations do not consist of non-overlapping groups but rather (self-)organize into other ecological patterns. We therefore present a mathematical framework for multi*scale* selection. This framework decomposes natural selection into two parts: *local selection*, acting *within* environments of a certain size, and *interlocal selection*, acting *among* them. Varying the size of the local environments subsequently allows one to measure the contribution to selection of each spatial scale. To illustrate the use of this framework, we apply it to models of the evolution of altruism and pathogen transmissibility. The analysis identifies how and to what extent ecological processes at different spatial scales contribute to selection and compete, thus providing a rigorous underpinning to eco-evolutionary intuitions.

## 1. Introduction

Spatial structure is the rule, rather than the exception, in biological populations. Spatial population structure may reflect heterogeneities in the abiotic environment, such as resource availability, but can also arise from self-organization through ecological interactions between individuals [1]. Examples span a large range of scales. Many bacteria live in biofilms that are highly heterogeneous [2–4] and in which interactions between bacteria are often limited to a range of a few micrometres [5–7]. At the same time, bushes growing in semi-arid areas form intricate vegetation patterns that span tens to hundreds of metres [8,9].

Because spatial population structure determines which organisms interact and compete, it is a key factor shaping evolution. A classical example is the evolution of altruism: behaviour that decreases an individual’s own fitness, but increases the fitness of its interaction partners [10,11]. It has long been recognized that a non-arbitrary interaction structure is necessary for altruism to evolve, so that the behaviour of altruistic individuals preferentially benefits other altruists [10,12–14]. Such interaction structure can arise naturally if ecological interactions and reproduction are local, and dispersal is limited, which leads to spatial assortment such that altruistic individuals are generally near other (related) altruists [3,15–19].

Classical work on the effect of spatial structure on natural selection often focused on populations that are subdivided into distinct groups, e.g. trait-groups within which interactions among organisms are compartmentalized for periods of time [12,20–23]. Selection is then considered at two levels, *within* and *between* groups, and the selection pressures at these two levels can be quantified [20]. Crucially, selection pressures within and between groups are not necessarily aligned. In the case of altruism, for example, selection within groups tends to favour selfish behaviour (also called cheating or defecting), while selection between groups promotes altruism [12,24–26].

Many biological populations, however, are (self-)structured in space but not subdivided into distinct groups within which most of the social interactions take place [1]. In the absence of biologically significant groups, application of standard group-selection theory is not straightforward and often not meaningful. Nevertheless, as in group-structured populations, selection pressures acting within local environments may differ from those observed at the scale of the whole population [27–30]. Returning to the example of altruism: cheaters may locally out-compete altruists even if, owing to emergent spatial patterning, altruism is favoured in the population as a whole [15,19,29]. While such a multi*scale* perspective on spatially structured populations seems analogous to the multi*level* perspective on group-structured populations, a formal treatment that defines and quantifies selection at each spatial scale is currently lacking.

Here, we therefore present a mathematically rigorous and highly general way to quantify natural selection at all spatial scales. A spatial decomposition of selection is derived that splits global selection into a local component, which describes the average selection *within* local environments, and an interlocal component, which describes the selection *among* these environments. By varying the size of the local environments the contribution to selection of each scale can be measured. To illustrate the use of this method, we apply it to two computational models of the evolution of traits known to be affected by spatial structure: altruism and pathogen transmissibility. We show how the spatial decomposition of selection captures the contribution to selection of ecological processes and patterns unfolding at different scales and enables a precise understanding of phenomena such as self-shading [31,32]. In the Discussion, we describe how the multiscale framework compares with and complements other mathematical approaches to analyse (models of) evolution in spatially structured populations, such as the use of moment equations [27,33–38], the theory of Markov processes [15,39,40], and population genetic approaches [41–46].

## 2. Results

### (a) A spatial decomposition of selection

Consider a spatially structured population of individuals that differ with respect to some phenotype *ϕ*. This could be a quantitative trait value (e.g. an individual’s investment in altruistic behaviour) or an indicator variable that is 1 for individuals that display a certain phenotype (e.g. altruism) or possess a certain gene, and 0 for those that do not. We are interested in the evolution of the mean value of *ϕ* over time. Over 50 years ago, George R. Price derived a highly general mathematical description of evolutionary change, showing that the change in mean value of any characteristic *ϕ* over a given time interval due to selection is equal to the covariance between *ϕ* and the relative fitness *w* among individuals [47]. This covariance, called the *selection differential* and denoted by *S*, provides a general measure of the strength and direction of natural selection.

**Figure 1.**
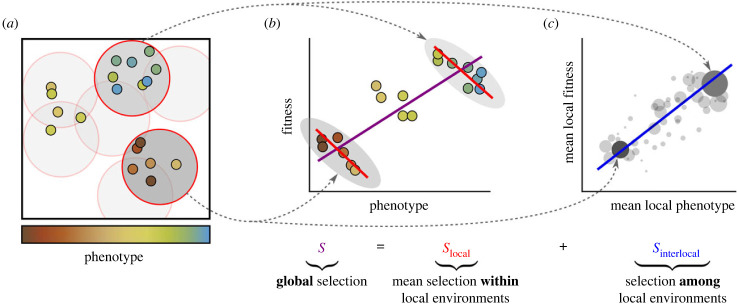
Illustration of the spatial decomposition of selection. (*a*) Spatially structured population of individuals that differ in some phenotypic characteristic. Local environments are defined as circular areas with a given radius. (*b*) Example of global and local selection pointing in different directions. The covariance between phenotype and fitness *within* all local environments is negative (i.e. local selection is negative), as evident from the negative slopes of the red regression lines; nevertheless, the global covariance between phenotype and fitness is positive (i.e. global selection is positive), as apparent from the positive slope of the purple regression line. This is an example of Simpson’s paradox. (*c*) The negative local selection is counteracted by a positive covariance between the mean phenotype and mean fitness of local environments (see the blue regression line). Local environments are weighted by their population density and mean fitness (size of points). This covariance represents the selection *among* environments, i.e. the interlocal selection.

Price’s selection differential describes the effect of selection at the scale of the whole population. In spatially structured populations, however, this may fail to capture the whole story. For example, consider the hypothetical population in figure 1*a*. Here, at the global scale, the covariance between phenotype and fitness is positive (purple regression line in figure 1*b*), yet if the analysis is restricted to individuals within smaller-scale local environments (circles in figure 1*a*), it is invariably negative (red lines in figure 1*b*). Counterintuitively, in such cases, the effect of natural selection is to reduce the mean of *ϕ* in every local environment, while driving it up globally; a spatial Simpson’s paradox [25,48].

To quantify selection at these smaller scales, we first need a mathematically rigorous definition of local environments. Here, we simply define local environments as circular areas (discs) with a given radius *r* (see Methods for a more general definition based on a *kernel function*). Note that local environments may overlap and that they are not necessarily centred on individuals (figure 1*a*). To quantify the effect of natural selection within a local environment, we can now define the *local selection differential* (LSD; see Methods) as the covariance between phenotype and relative fitness measured in the sub-population present within that local environment. For any given point in space, the LSD at scale *r* is subsequently defined as the LSD of the local environment of scale *r* centred on this point.

Using this formal definition of selection within local environments, for any scale *r*, we can derive the following spatial decomposition of the selection differential of the full population:

2.1 $$\begin{array}{rl} S & =mean(\mathrm{LSD})+Cov(\mathrm{local}\;\mathrm{mean}(\phi ),\mathrm{local}\;\mathrm{mean}(w))\\ & \equiv S_{\mathrm{local}}(r)+S_{\mathrm{interlocal}}(r), \end{array}$$

which is the central result of this article (figure 1). The first term, *S*_local_(*r*), is defined as the average of the LSD over all local environments, which measures the selection observed on average *within* local environments of scale *r*. It hence represents the *local* component of selection. The second term, *S*_interlocal_(*r*), is the covariance between local mean phenotype and local mean fitness, which measures to what extent local environments with a higher mean phenotype tend to have higher mean fitness. This term can therefore be interpreted as the *interlocal* component of selection and represents selection *among* environments of scale *r*.

It bears stressing that the boldface mean and covariance in equation (2.1) are calculated over an infinite number of overlapping local environments. This is a crucial difference with standard group-selection theory, which deals with populations subdivided into a finite set of non-overlapping groups. To properly calculate this mean and covariance, local environments have to be weighted according to the local population density. A detailed derivation is provided in the Methods.

The measure of local selection, *S*_local_(*r*), captures the effect of all ecological processes that produce fitness differences among individuals within local environments of size *r*. In other words, it incorporates all eco-evolutionary mechanisms operating at length scales smaller than or equal to *r*. To identify how a *specific* length scale *r* contributes to selection, we should ask how the local selection component changes if we slightly expand the scale of the local environments from *r* to *r* + d*r*. That is, the *contribution to selection* of scale *r*, which we denote by *s*(*r*), is captured by the derivative of *S*_local_(*r*) with respect to *r*. Assuming that no two individuals can be at the exact same position in space, so that *S*_local_(0) = 0, we can then write

2.2 $$S=\int_{0}^{\infty }s(r^{\prime })dr^{\prime },\;S_{\mathrm{local}}(r)=\int_{0}^{r}s(r^{\prime })dr^{\prime }\;\mathrm{and}\;S_{\mathrm{interlocal}}(r)=\int_{r}^{\infty }s(r^{\prime })dr^{\prime },$$

which decomposes *S*, *S*_local_(*r*) and *S*_interlocal_(*r*) into contributions of different scales.

Equation (2.2) exhibits several general properties of the local and interlocal selection components. If the local environments are made ever smaller (*r* → 0), *S*_local_(*r*) approaches 0 and *S*_interlocal_(*r*) approaches *S*: very small local environments contain at most one individual, and hence lack variation in phenotype and fitness, which implies that local selection must vanish. If environments are instead made larger and larger, then *S*_local_(*r*) approaches *S*, as it should: large enough ‘local’ environments capture the global population dynamics. In the same limit, *S*_interlocal_(*r*) approaches 0, because in large environments the local means of phenotype and fitness approach the global mean, eliminating variation between environments and hence selection between them.

Conversely, if *S*_local_(*r*) deviates substantially from *S* for some value of *r*, we can conclude that selection within environments of scale *r* is not representative of the global dynamics, and hence that spatial patterns relevant to selection must exist at scales approximately equal to *r* or larger. The same conclusion can be reached from the perspective of *S*_interlocal_(*r*). If *S*_local_(*r*) deviates significantly from *S*, then *S*_interlocal_(*r*) deviates from 0 (since *S*_interlocal_(*r*) = *S* − *S*_local_(*r*)). Because *S*_interlocal_(*r*) is defined as the covariance between local mean phenotype and local mean fitness, a significant deviation from 0 requires a significant *variance* in local mean phenotype and local mean fitness among local environments of scale *r*. This implies that the population shows spatial assortment at a scale of the order of *r* or larger.

To illustrate the use of the multiscale selection framework (equations (2.1) and (2.2)), we apply it to two models of classical eco-evolutionary feedback: (i) the evolution of altruism, and (ii) the evolution of pathogen transmissibility.

#### (i) Example I: evolution of altruism aided by self-organizing colonies

The first model, on the evolution of an altruistic trait, was first described and analysed in a recent publication [49]. There, it was studied primarily from the perspective of multi*level* selection; here, we will briefly describe its dynamics and then analyse it from a multi*scale* perspective. The fact that this model can be analysed from both perspectives makes it ideally suited to illustrate their differences.

**Figure 2.**
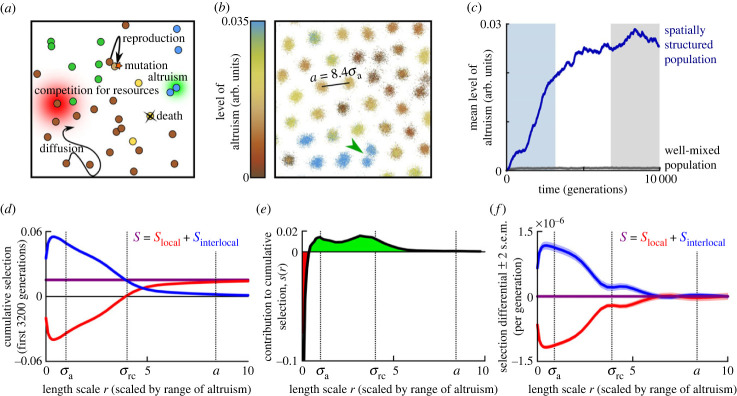
Evolution of altruism. (*a*) Cartoon illustration of the model. Both resource competition and altruism are local processes. The range of resource competition, *σ*_rc_, is larger than the range of altruism, *σ*_a_. (*b*) Snapshot of part of the simulation plane (see electronic supplementary material, movie S1 for dynamics). The hexagonal lattice constant of the emerged colony pattern is *a* = 8.4*σ*_a_. The green arrow indicates a colony fission event. (*c*) Mean level of altruism over time, in a population that is well-mixed (grey) or spatially structured (blue). (*d*) Cumulative selection (purple) and its spatial decomposition (red and blue) as a function of the length scale *r* of local environments, measured over the first 40 000 simulation time steps (3200 generations; blue-shaded area in (*c*)). (*e*) Contribution to cumulative selection of different length scales measured over the first 40 000 simulation time steps (calculated as the derivative of *S*_local_(*r*) in (*d*) with respect to *r*; see equation (2.2)). The red area indicates a negative contribution to selection, green a positive contribution. (*f*) Spatial decomposition of selection differential *S* at evolutionary equilibrium. *S*_local_(*r*) and *S*_interlocal_(*r*) were calculated as averages over the last 40 000 simulation time steps (3200 generations) of the simulation (grey-shaded area in (*c*)).

The model considers a population of individuals in two-dimensional space that reproduce, die, move around slowly, and locally compete for resources (figure 2*a*; electronic supplementary material, text). Individuals are characterized by a continuous trait that represents their investment in altruism. Upon reproduction, this trait value is passed on from parent to offspring, at which time mutations are introduced with small probability. Altruistic behaviour directly reduces an individual’s reproduction rate, but benefits all individuals in the local social environment of the altruist. The effects of altruism and resource competition both depend on the distance between organisms, such that an individual that is close to others benefits more from their altruistic action, but also experiences stronger competition (see electronic supplementary material, text for details). The distances beyond which the effects of altruism or resource competition become weak—the ‘interaction scales’—are denoted by *σ*_a_ and *σ*_rc_. Units are chosen such that the range of altruism *σ*_a_ equals 1 and time is measured in generations, where one generation is defined as the mean lifespan of an individual.

If the interaction scale of resource competition, *σ*_rc_, is sufficiently larger than the scale of altruism, *σ*_a_, the model population shows intriguing self-organized behaviour (figure 2*b*; electronic supplementary material, movie S1): a Turing-like instability results in a hexagonal pattern of distinct colonies that display Darwinian dynamics of their own. In colonies with a high mean level of altruism, the density of individuals is high because they all benefit from the altruism of colony members. Over time, however, the level of altruism within a colony declines because mutants with lower levels of altruism are selected (‘defectors’ or ‘cheaters’)—a within-colony ‘tragedy of the commons’. This decline eventually results in the demise of the colony, which is subsequently replaced by a newly formed colony that originates from the binary fission of one of the surrounding colonies (see arrowhead in figure 2*b*; electronic supplementary material, movie S1). Crucially, colonies with a higher mean level of altruism are more likely to ‘reproduce’ by binary fission. (For an in-depth analysis of the colony dynamics, see [49].) In earlier mechanistic models that show Darwinian dynamics at multiple levels, the group structure and reproduction are generally imposed either directly as part of the specification of the model (e.g. [12]) or by an assumed ecological scaffold [50]; here they instead emerge spontaneously from the ecological interactions among individuals.

The simulations are initialized with individuals that do not invest in altruism. But, over the course of the simulation, the mean level of altruism increases systematically (figure 2*c*). By contrast, if we destroy the self-organized pattern by mixing the population (i.e. randomly assigning positions to individuals) at every time step, altruism does not evolve at all (figure 2*c*). The spatial patterns that emerge from local reproduction and ecological interactions are hence crucial for the evolution of altruism [49], consistent with previous modelling work [15–17,51,52].

The behaviour of the emergent colonies suggests that natural selection acts both at the level of individuals and at the level of the colonies. Therefore, it is natural to analyse the model from a multi*level* perspective. This was done in [49] to confirm that the within-group selection on altruism is negative, but that this is offset by a positive among-group selection. Here, however, we analyse and quantify the role of the spatial patterns by applying the multiscale selection framework (equation (2.1)). We first focus on the initial part of the simulation, when the mean level of altruism increases (the first 3200 generations, shaded in blue in figure 2*c*). To measure the effect of natural selection we calculated the selection differential *S* over each simulation time step. The sum of these selection differentials represents the contribution of selection to the total change in the population mean level of altruism over this part of the simulation; we call it the *cumulative* effect of selection. Each time step, we also used equation (2.1) to decompose *S* into the components *S*_local_(*r*) and *S*_interlocal_(*r*), for many values of the scale of local environments *r*. (See Methods; also, see electronic supplementary material, text for efficient methods to compute *S*_local_(*r*) and *S*_interlocal_(*r*).) Subsequently we calculated the cumulative values of *S*_local_(*r*) and *S*_interlocal_(*r*) by summing up their values for all time steps. These cumulative values measure the contributions of local and interlocal selection to the total change in the population mean level of altruism over the initial part of the simulation.

The result is plotted in figure 2*d*. The (cumulative) values of *S*_local_(*r*) and *S*_interlocal_(*r*) observed for small and large values of *r* reflect the general properties of the spatial decomposition discussed above (see equation (2.2)). In particular, in the limit of large *r*, the value of *S*_local_(*r*) (red line) converges to the global selection differential *S* (indicated by the purple horizontal line), and *S*_interlocal_(*r*) (blue line) converges to zero. The cumulative value of *S* is significantly positive, confirming that, globally, the selection on altruism is positive during this initial part of the simulation. The spatial decomposition, however, reveals that, for *r*-values up to approximately the interaction range of resource competition *σ*_rc_ (which in these simulations is equal to four times the interaction range of altruism *σ*_a_), the local selection *S*_local_(*r*) (red line) is in fact *negative*. This negative local selection is compensated by strongly positive interlocal selection *S*_interlocal_(*r*) (blue line). These results capture and formalize the narrative suggested above: within local environments, individuals with a lower level of altruism have the upper hand because they benefit from the altruistic behaviour of others nearby while paying lower costs; but local environments in which the mean level of altruism is low also tend to have low mean fitness, i.e. selection *among* local environments favours higher levels of altruism. The same result is also found by considering the contribution of each length scale to selection, *s*(*r*) (figure 2*e*): small length scales contribute negatively to selection, whereas larger length scales contribute positively.

These differences between local and interlocal selection only arise in spatially structured populations; the same analysis on the well-mixed population yields none of these interesting effects (electronic supplementary material, figure S1).

Based on the observed tragedies of the commons within colonies, one might expect that the scale associated with negative selection on altruism is tied to the size of single colonies. This is indeed the case. The typical distance between the centres of neighbouring colonies is given by the lattice constant of the emerging hexagonal colony pattern, *a* = 8.4*σ*_a_ (figure 2*b*). Local environments with a scale (radius) below *r* ≈ *a*/2 typically contain individuals from one colony only, and selection within such small environments should therefore be negative on average. Larger environments are able to encompass competing colonies, and hence selection within large environments should be positive on average. This expectation is borne out: figure 2*d* demonstrates that *S*_local_(*r*) first becomes positive when *r* exceeds approximately *a*/2, which is approximately *σ*_rc_.

However, the results also indicate that the selection against altruism is at its strongest at a much smaller scale *r* ≈ *σ*_a_/2 (figure 2*d*). In line with this, measurements of *s*(*r*) (figure 2*e*) reveal that only very small length scales of $r⪅\sigma_{a}/2$ contribute negatively to selection. This indicates that colonies are not homogeneous: even within a single colony we observe assortment of individuals with different investment in altruistic behaviour, and this assortment contributes positively to the selection of altruism. This is understandable: individuals that are very close together experience a similar level of altruism and competition. In very small local environments, cheaters hence must have an advantage over altruists because they pay lower costs but profit equally. But once the diameter of local environments exceeds the range of altruistic interactions, individuals within the same local environment may experience different levels of altruism, and the effects of these heterogeneities start to contribute to local selection.

So far, we have considered the first part of the simulation, in which altruism was under positive selection. Later in the simulations, once the mean level of altruism has stabilized, we should expect the global selection differential to equal zero on average because no directional selection remains. To average out fluctuations arising from the stochastic dynamics in the finite population, we analyse the selection differential over the last 3200 generation times (grey-shaded area in figure 2*c*), and indeed find that, over this period, *S* ≈ 0 (purple line in figure 2*f*). Nevertheless, selection within local environments remains negative (red curve), and this negative local selection is counteracted by positive interlocal selection (blue curve).

Looking further into which spatial scales are relevant for selection, we see that the scale at which *S*_local_ converges to *S* and *S*_interlocal_ converges to 0 is close to the lattice constant *a* (figure 2*d*,*f*). This indicates that local environments that are large enough to contain multiple neighbouring colonies already capture most processes and patterns contributing significantly to global selection; larger-scale patterns, such as the clear assortment at the colony level (see figure 2*b*; nearby colonies tend to have similar mean altruism levels), appear to have a negligible effect.

In conclusion, the multiscale selection framework allows one to mathematically show that local selection for altruism is negative, but that this is compensated by positive interlocal selection. It furthermore provides a way to quantify how specific length scales contribute to selection, thus revealing which patterns and processes are significant for natural selection. Specifically, it shows that the assortment within colonies is relevant to selection, whereas assortment at the colony level beyond neighbouring colonies is not.

### (b) Example II: evolution of pathogen transmissibility in an SI model

As a second example, we consider the evolution of the transmission rate of an endemic pathogen in a spatially structured population of host individuals. We use a classical individual-based epidemiological model of the SI type, i.e. describing a population consisting of susceptible (S) and infected (I) organisms; see also e.g. [31,33,38,53,54] for similar implementations. We present this model because earlier studies point to an incongruity between local an global selection pressures in this class of models, with local selection favouring ever-increasing transmission rates but global selection favouring pathogen restraint [31,33,36]. We will demonstrate that the multiscale decomposition can rigorously define, demonstrate, and quantify this phenomenon, thus providing a clear example of the use of the multiscale analysis.

**Figure 3.**
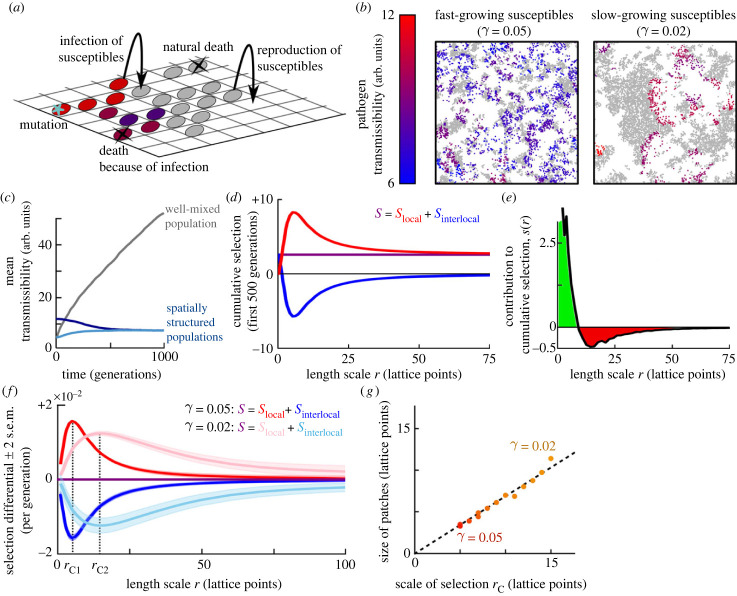
Evolution of pathogen transmissibility. (*a*) Cartoon illustration of the model. (*b*) Snapshot of part of the simulation lattice for two different values of the reproduction rate of susceptible individuals, *γ*. Susceptible individuals are plotted in grey, infected individuals are coloured based on the transmissibility of the pathogen they carry. See electronic supplementary material, movies S2 and S3 for dynamics. (*c*) Mean transmissibility of the pathogen over time in populations that are well-mixed (grey) or spatially structured (blue) for default parameter settings (*γ* = 0.05). Two spatially structured simulations are shown which were initialized with different transmissibility values. Time is measured in generations, with generation time defined as mean lifespan of susceptible individuals. (*d*) Spatial decomposition of cumulative selection in the default simulation (*γ* = 0.05, initial transmissibility = 5, lighter blue line in *c*) in the early part of the simulation, where selection on transmissibility is positive (*S* > 0). *S*, *S*_local_ and *S*_interlocal_ were summed over the first 10 000 simulation time steps (500 generations). (*e*) Contribution of varying length scales to the cumulative selection over the first 500 generations, calculated as the derivative of *S*_local_(*r*). (*f*) Spatial decomposition of the selection differential at evolutionary equilibrium. For both values of *γ*, *S*_local_ and *S*_interlocal_ were now calculated as the mean value over 10 000 simulation time steps between time = 9500 and 10 000 generations. We define the critical scale of selection, *r*_C_, as the length scale at which the contribution to selection switches from positive to negative (i.e. where *s*(*r*) = d*S*_local_/d*r* switches sign). (*g*) Critical scale of selection, *r*_C_, plotted against size of the emerged patterns for different values of the susceptible reproduction rate *γ*. Pattern size was determined using the pairwise correlation function (see electronic supplementary material, text).

In the model, host individuals live on a two-dimensional square simulation lattice (figure 3*a*). They are either susceptible to infection, or infected. Susceptible individuals reproduce asexually, in which case the offspring is placed on a neighbouring lattice site. Each lattice site can hold at most one individual; susceptible individuals therefore locally compete for space. Infected individuals do not reproduce, and they die at a higher rate than susceptible individuals. The pathogen is transmitted from infected to neighbouring susceptible individuals at a rate that varies among pathogen variants. We consider the evolution of this pathogen transmissibility. For simplicity, each infected individual is assumed to carry a single pathogen strain, and mutations instantaneously change the transmissibility of all pathogens within a single infected host (i.e. newly arising pathogen variants rapidly sweep the within-host pathogen population). Details on the model implementation are provided in the electronic supplementary material.

After initialization, the simulated population quickly self-organizes into ecological patterns: the infection chases patches of susceptible individuals in wave-like structures (figure 3*b*; electronic supplementary material, movies S2 and S3). These patterns strongly influence the evolution of pathogen transmissibility (figure 3c): if pattern formation is prevented by constantly mixing the population, pathogens with ever-increasing transmissibility are selected because pathogens with higher transmissibility spread faster among the available susceptible hosts. In spatially structured populations, however, the mean transmissibility eventually stabilizes at a value independent of initial pathogen transmissibility (blue lines in figure 3*c*). This is explained by a feedback between evolution and the ecological patterns: pathogen strains shape their local environment, and this environment in turn affects the strain’s local fitness [1,28,55]. Specifically, pathogens with very high transmissibility rapidly deplete the susceptible hosts in their vicinity and are then left with little opportunity to spread, locally resulting in low average pathogen fitness. By contrast, more prudent pathogens shape their environment in such a way that sufficient susceptible hosts remain available to allow the infection to continue spreading [53,56]. Such processes have been referred to as ‘self-shading’ [31,32].

We use the multiscale selection framework to quantify and formalize this self-shading. First, consider the first 500 generations. In the default simulation (initial transmissibility = 5, shown as the lighter blue line in figure 3*c*), the transmissibility increases over this period, suggesting that it is under positive selection. Indeed, the cumulative selection, calculated as the sum of *S* over all computational time steps in the first 500 generations, is significantly positive (figure 3*d*, purple line). The spatial decomposition of *S* for varying length scales *r*, however, provides additional insight into the forces shaping this positive selection. As expected, at very small values of *r*, *S*_local_ approaches 0, because very small local environments contain at most one individual and hence lack the variance in transmissibility and fitness required for selection. It follows that *S*_interlocal_ approaches *S*. Within local environments of all scales *r* > 0, however, selection clearly favours pathogens with high transmissibility (*S*_local_(*r*) > 0, red curve in figure 3*d*). At scale *r* ≈ 10 lattice points, this local positive selection is much stronger than the overall selection, but it is partly counteracted by negative interlocal selection (blue curve). This negative interlocal selection captures the effect that, in local environments populated by pathogens with high average transmissibility, susceptible host availability and hence pathogen spread tend to be limited.

To determine how interactions on each length scale contribute to selection, we study the derivative of *S*_local_(*r*), *s*(*r*) (figure 3*e*). It reveals that the overall positive selection on transmissibility is only driven by processes occurring at the smallest scales (*r* < 5 lattice points). Larger scales actually contribute negatively to the selection on transmissibility, reducing the positive selection on this pathogen trait. These results underline the conclusions based on the calculation of *S*_local_(*r*) and *S*_interlocal_(*r*), namely that selection favours pathogens with high transmissibility at small spatial scales, but favours pathogen restraint at larger spatial scales. Thus, they support the intuitive idea that self-shading can play an important role in pathogen evolution in spatially structured environments.

Very similar ecological patterns are found in an alternative simulation that was initiated with a higher transmissibility value (dark blue line in figure 3*c*), with one major difference: global mean selection is now negative (*S* < 0) and hence the curves are shifted vertically (electronic supplementary material, figure S2). Otherwise, how different length scales contribute to selection remains similar: local selection on transmissibility is positive, interlocal selection negative, and only the smallest length scales contribute positively. By contrast, in well-mixed populations none of these patterns is found: selection on transmissibility is strongly positive and fully captured by *S*_local_(*r*) as long as the local environments are large enough to contain representative samples of the population’s phenotypic variation (electronic supplementary material, figure S3).

When the population has reached evolutionary equilibrium (figure 3*f*, dark curves), the global selection differential *S* is insignificant as expected (because the global mean transmissibility no longer changes), but within local environments selection still favours pathogens with high transmissibility (*S*_local_(*r*) > 0), and this effect is counteracted by negative interlocal selection (*S*_interlocal_(*r*) < 0).

We can now further explore how the spatial patterns affect the eco-evolutionary process. The size of the spatial patterns that emerge in the population depends on several model parameters [53], including the reproduction rate of the susceptible hosts: lower reproduction rates result in larger patterns (figure 3*b*). To demonstrate how these larger patterns are reflected in *S*_local_ and *S*_interlocal_, we repeated the analysis with a lower susceptible reproduction rate (right panel in figure 3*b*, pink and light-blue lines in figure 3*f*). Evidently, the curves representing *S*_local_(*r*) and *S*_interlocal_(*r*) are stretched towards larger scales. This reflects that the spatial scales relevant to selection depend on the size of the patterns in the population: after all, selection for pathogen restraint can only be observed if the local environments in which selection is measured are large enough to cover multiple patches.

To verify this relationship between multiscale selection and spatial pattern size, we define the critical scale of selection, *r*_C_, as the length scale at which the contribution of length scales to selection, *s*(*r*), switches sign (figure 3*f*), such that scales smaller than *r*_C_ contribute positively to selection, and scales larger than *r*_C_ contribute negatively. This critical scale of selection is an emergent property of the eco-evolutionary dynamics. By repeating the analysis for a range of susceptible growth rates, we find that the critical scale of selection is proportional to the size of the emergent patterns (figure 3*g*). Hence, the *S*_local_ and *S*_interlocal_ curves, and specifically the critical scale of selection, *r*_C_, capture the length scale of the spatial structures that are relevant for natural selection.

In conclusion, this second example illustrates that the multiscale selection framework can be used to measure and quantify self-shading in eco-evolutionary processes. It furthermore allows the identification of the scale(s) of population structures relevant to natural selection.

## 3. Discussion

We have presented a new, multiscale selection framework that can be used to analyse evolution in spatially structured populations. The framework is based on a spatial decomposition of selection (equations (2.1) and (2.2)) that quantifies local and interlocal selection for any spatial scale. It was previously understood that the direction of natural selection at local and global scales can differ, and verbal arguments have been made based on analogies with group selection, but no formal framework existed that allowed one to verify and quantify such effects. This work provides such a formalism.

Two example models illustrated how this framework can be used to measure the contribution to selection of processes and patterns at varying scales, and thus to identify the spatial scales relevant to natural selection. While the two models describe different biological systems and phenomena, in both cases the direct interest of and individual runs counter to the interest of others in its local environment; therefore, the phenotypes that perform well in local environments may not perform well in the population as a whole. The examples—on the evolution of altruism and of pathogen transmissibility—were chosen because they are among the best-known examples of eco-evolutionary feedback between spatial patterns and natural selection [29,38], and because this feedback has also been confirmed experimentally. For instance, increased population viscosity facilitates the evolution of altruistic public good production in lab populations of *Pseudomonas aeruginosa* [57,58], and several experiments have shown that increased host population viscosity and/or localized pathogen spread select for lower virulence in an insect larval virus [32] and bacterial viruses [59,60]. The effect of spatial structure on natural selection is, however, by no means limited to these two examples. It was first described in models of catalytic hypercycles of self-replicating molecules, which give rise to self-organized rotating spirals that select for higher death rates of the individuals constituting these spirals [61,62]. Since then, many other examples have been described [1,28], including anti-competitor toxin production in bacteria [63–67]. Applying the multiscale selection framework to such other examples could lead to new insights in these systems as well.

In principle, the multiscale analysis can also be applied to experimental data to study real populations. Because the selection differential of the Price equation is just the covariance between trait value and fitness, it can and has been estimated from sampled data (e.g. [68]). If in addition information is available on the position of sampled organisms, the local and interlocal selection components *S*_local_ and *S*_interlocal_ can also be estimated. However, to estimate *S*_local_ and *S*_interlocal_ with high precision, a large sample size is required, especially if selection is weak. While such data are easily obtained in simulation studies, it remains to be seen whether this is feasible for observational or experimental studies.

Even if the statistical power is too low to reliably calculate local and interlocal selection for all spatial scales, the multiscale framework can provide insights into potential effects of spatial structure in data. As our analysis shows, natural selection in local environments can substantially differ from the selection in the global population, even if an average is taken over all local environments. Evolutionary studies based on local observations can hence provide an incomplete picture of the evolutionary dynamics in the global population. The framework presented here provides a way of determining whether a collection of local sampling areas is representative of the whole population under selection. Namely, if the local sampling areas are large enough to capture all spatial structures and processes relevant to selection, interlocal selection vanishes and the mean local selection differential (i.e. as measured within sampling areas) is equal to the global selection differential. Specifically, this means that there should be no covariance between mean phenotype and mean fitness among local environments (i.e. *S*_interlocal_ = 0). A significant correlation between local mean phenotype and local mean fitness is hence a strong indication that larger structures exist in the population that contribute to natural selection.

The multi*scale* decomposition of selection of equation (2.1) is analogous to the multi*level* decomposition into within- and between-group components derived by Price in 1972 [20]. However, it differs from it in fundamental ways and as such provides a different perspective on the effects of (spatial) structure on selection and applies to different systems. The multilevel framework requires that distinct and non-overlapping groups are defined. While one can always do so—in countless arbitrary ways—the multilevel decomposition is only meaningful if the groups represent biologically relevant clusters of social interactions. Crucially, in some systems such clusters do not exist, in particular if the spatial patterns are variable in time and space, such as the patches in the model of pathogen transmissibility; and in such cases the multiscale approach is more appropriate. If distinct groups *can* be easily recognized, the multilevel selection framework can provide a quantification of the selection pressures acting at different levels of organization; but even then the multiscale approach may provide additional information. Many classical models of group selection assume, implicitly or explicitly, that conditions within groups are homogeneous; that social interactions exclusively take place within groups; and that these interactions are binary and transitive (i.e. individuals do not interact to varying degree, and if A interacts with B and B with C, then A interacts with C). Spatially explicit models and real biological systems are unlikely to obey such assumptions, and in that case the multiscale approach may reveal relevant spatial structure within or among groups that an exclusively multilevel approach would overlook. This point is illustrated by the altruism model. Because in that model the emergent colonies can readily be recognized, it is possible to apply multilevel theory to quantify the within-colony tragedy of the commons and the among-group selection favouring altruism [49]; but by design this approach is blind to the relevant spatial structure within groups that was revealed by the multi*scale* approach.

It is worth stressing that the multiscale decomposition of the selection differential is highly general. Because the analysis is based on the selection differential of the Price equation, which quantifies the effect of selection over a time interval from *t*_1_ to *t*_2_, the only assumptions required to calculate it are that at these times each individual has a well-defined trait value, that each individual in existence at time *t*_2_ has one or more ancestors at time *t*_1_, and that each individual in the population has a well-defined position at time *t*_1_. In the two examples above, the space was two-dimensional and Euclidean, but the approach works for any number of dimensions and can straightforwardly be extended to other geometries, such as the surfaces of spheres.

Over the past decades the interest in eco-evolution in spatially (self-)structured populations has increased sharply. This has also spurred important progress in the mathematical analysis of evolution in spatially structured populations [1]. We here describe three important lines of work and discuss how the multiscale selection framework differs from and may complement these analyses.

First, population geneticists have long been concerned with understanding and predicting the fate of neutral, deleterious and advantageous alleles in spatially structured populations [41]. Using metapopulation models of connected demes, classical results have been obtained for fixation probabilities (e.g. [42,43,46]), effective population size (e.g. [43,44]), and coalescence times (e.g. [45]). While such studies have yielded powerful tools to analyse (genomic) data from spatially structured populations, their focus differs fundamentally from this study. In particular, theoretical population genetics studies are often concerned with the long-term fate of alleles with given fitness effects. Because they generally do not incorporate (local) frequency-dependence or feedbacks between ecological and evolutionary dynamics, the results cannot (yet) be easily extended to questions like the ones addressed here.

Second, progress has been made by applying and expanding the theory of Markov processes to prove mathematical theorems that hold for a wide class of models of spatially structured populations [39,40,69]. These results range from fundamental relations between measures of evolutionary success of alleles [39], such as their average fitness or expected frequency, to general expressions for mutation-fixation probabilities applicable to populations of any size and interaction structure [40]. These successes notwithstanding, the results are so far limited to populations with a fixed population size and interaction structure; that is, an individual at a given position interacts with a fixed number of individuals at a predetermined set of other sites. Hence, these analyses cannot yet incorporate the feedback between emergent spatial structure and natural selection characteristic of eco-evolutionary dynamics.

Third, several authors have shown how the dynamics of grid-based models can be analysed using spatial moment equations, which express changes in e.g. the mean (first moment) or variance (second moment) of a trait under selection in terms of correlations between the states of neighbouring sites (e.g. [27,29,30,33–38]). Provided the hierarchy of moment equations is closed using pair approximation or another closing scheme, this approach can be used to predict, among other things, invasion conditions and evolutionarily stable strategies [34]. Moreover, even without such approximations, the terms and structure of the moment equations themselves can provide insight into components of the evolutionary process [1]. In particular, the differential equation for the first moment (the mean)—essentially a continuous-time Price equation—tends to feature terms describing the effects of both local and global forces on the mean value of the trait [38], which can then be analysed to derive how model parameters influence the balance between these local and global effects. While this is reminiscent of the spatial decomposition presented here, there are fundamental differences between these two types of decomposition. In spatial moment equations, the scales included in the analysis are defined *a priori*. Although multiple scales can be introduced [70], usually only correlations between neighbouring grid cells are considered. ‘Local’ effects then only extend to effects of the direct neighbourhood, while all other effects are captured in ‘global’ terms, and the decomposition is therefore agnostic of the actual scales of emerging spatial structures. Also, the local (co)variances that appear in this work are defined in terms of the neighbourhoods surrounding individuals of a given type rather than in terms of the trait values of individuals found in local environments of a particular *size*, as in our framework. This underscores that our approach is fundamentally different and complementary to spatial decompositions resulting from spatial moment equations.

While the multiscale decomposition of the selection differential is new and uniquely untangles local and interlocal selection in spatially structured populations, it is not the only, nor the only correct way the selection differential can be decomposed. Next to the distinct-groups multilevel approach and the spatial moments approach discussed above, the selection differential can also be decomposed into terms that capture the effect of an individual’s own character on its fitness, and the effect of its environment (the contextual analysis approach to multilevel selection) [71], or in terms that capture the effect of an individual's behaviour on its own fitness and on the fitness of related interaction partners (the inclusive fitness framework) [30,72–76]. Each decomposition of the selection differential tells a potentially new story about the underlying mechanisms driving evolution. The multiscale selection framework presented here is an addition to the toolbox available to address complex eco-evolutionary questions.

## 4. Methods

Below, we derive equation (2.1). Full specifications of the simulation models, as well as computational details on the calculation of the two terms of equation (2.1), are provided in electronic supplementary material, text.

### (a) Background: definition of the selection differential *S*

Consider a population in space that at some time *t* consists of *n* individuals. Let *ϕ*_*i*_ be the phenotype of individual *i*, and *W*_*i*_ its fitness, defined as the number of offspring at some later time *t* + Δ*t*, including the individual itself if it survived over the time step Δ*t*. Price’s equation [47] now states that

4.1 $$\underset{\mathrm{change}\;\mathrm{in}\;\mathrm{mean}\;\mathrm{value}\;\mathrm{of}\;\phi }{\underbrace{\Delta \bar{\phi }}}=\underset{\mathrm{change}\;\mathrm{due}\;\mathrm{to}\;\mathrm{selection}}{\underbrace{\mathrm{Cov}(\phi ,w)}}+\underset{\mathrm{change}\;\mathrm{due}\;\mathrm{to}\;\mathrm{transmission}\;\mathrm{biases}}{\underbrace{\bar{w\Delta \phi }}},$$

where $w_{i}=W_{i}/\bar{W}$ is the relative fitness of individual *i* over the time interval (we use the common notation $\bar{c}$ to denote the population mean of characteristic *c*) and Δ*ϕ* captures biases in the transmission of phenotypic values from parents to offspring. Importantly, the first term of the Price equation reveals that the effect of selection on the mean phenotype is captured by the covariance between phenotype and fitness; this term is also called the *selection differential*. The analysis presented here focuses on the selection differential only; for a full version of the Price equation with overlapping generations, including transmission and survival-bias effects, see e.g. [77].

### (b) Measuring selection in a local environment: the local selection differential

For any point ***m*** in space, the local population density is defined as a conventional kernel density estimate

4.2 $$D(m;r)\equiv \sum_{i=1}^{n}K(m-x_{i};r).$$

Here, ***x****_i_* is the position of individual *i*. Its contribution to the density at position ***m*** depends on its distance to position ***m*** according to the kernel function *K*(***y***; *r*). The parameter *r* is the scale parameter (or band width) of the kernel function. In effect, the kernel function defines the local environments: it determines which organisms contribute to what extent to the environment around each point in space.

In this paper, we use disc-shaped kernel functions that include an individual in the local environment only if its distance to the environment’s midpoint is smaller than a given radius, *r*. Other reasonable choices for the kernel function include bi-variate normal or exponential distributions, which weigh individuals close to the focal point more heavily than those further away.

A proper kernel function is normalized (i.e. the integral of *K*(***y***; *r*) over space is equal to 1); it follows that

4.3 $$\int_{S}D(m;r)dm=n,$$

where $\int_{S}$ represents the integral over the entire space.

For any characteristic *c* of individuals, such as phenotype or fitness, we define the *local mean* at point ***m*** as

4.4 $${\left\{c;m,r\right\}}_{l}\equiv \frac{\sum_{i}K(m-x_{i};r)c_{i}}{D(m;r)},$$

which is the average of *c* over all individuals, weighted by the kernel function based on their position relative to ***m***. We will often write {*c*}_l_ as a shorthand for {*c*; ***m***, *r*}_l_ to avoid clutter.

Analogous to the selection differential in Price’s equation, we can now define the local selection around point ***m*** as the covariance between phenotype and relative fitness within the local environment, which we call the local selection differential (LSD):

4.5 $$\begin{array}{rl} S_{l}(m;r) & \equiv \frac{{\left\{\phi \;W;m,r\right\}}_{l}-{\left\{\phi ;m,r\right\}}_{l}{\left\{W;m,r\right\}}_{l}}{{\left\{W;m,r\right\}}_{l}}\\ & \equiv {\mathrm{Cov}}_{l}(\phi ,W/{\left\{W\right\}}_{l};m,r). \end{array}$$

Note that the LSD is equal to the local covariance between *ϕ* and local relative fitness (*W*/{*W*}_l_), i.e. the fitness of an individual relative to others in the local environment. Thus defined, it measures the effect of selection on the change in the local mean of *ϕ* at position ***m***, {*ϕ*; ***m***, *r*}_l_.

### (c) Decomposing the selection differential into local and interlocal selection

For any function over space *g*(***m***), we define the *spatial mean* as

4.6 $$\begin{array}{rl} {\left\langle g(m);r\right\rangle }_{s} & \equiv \frac{\int_{S}D(m;r)g(m)\;dm}{\int_{S}D(m;r)dm}\\ & =\frac{1}{n}\int_{S}D(m;r)g(m)dm. \end{array}$$

Note that this represents the average of *g*(***m***) over the complete space, but that each position ***m*** is weighted by the local density *D*(***m***; *r*). This weighting is equivalent to the weighting by group size in Price’s derivation of within- and between-group selection in a population consisting of distinct groups [20,21]. For readability, we will often write 〈*g*(***m***)〉_s_ for 〈*g*(***m***); *r*〉_s_. Conveniently, given the definitions of equations (4.4) and (4.6), the spatial mean of the local mean is simply the population mean, i.e. ${\left\langle {\left\{c\right\}}_{l}\right\rangle }_{s}=\bar{c}$.

Using these definitions, we can now derive the desired decomposition

4.7 $$\begin{array}{rl} S=\mathrm{Cov}(\phi ,w) & =\bar{\phi w}-\bar{\phi }\bar{w}\\ & ={\left\langle {\left\{\phi w\right\}}_{l}\right\rangle }_{s}-{\left\langle {\left\{\phi \right\}}_{l}\right\rangle }_{s}{\left\langle {\left\{w\right\}}_{l}\right\rangle }_{s}\\ & ={\left\langle {\left\{\phi w\right\}}_{l}-{\left\{\phi \right\}}_{l}{\left\{w\right\}}_{l}\right\rangle }_{s}+{\left\langle {\left\{\phi \right\}}_{l}{\left\{w\right\}}_{l}\right\rangle }_{s}-{\left\langle {\left\{\phi \right\}}_{l}\right\rangle }_{s}{\left\langle {\left\{w\right\}}_{l}\right\rangle }_{s}\\ & ={\left\langle {\mathrm{Cov}}_{l}(\phi ,w;m,\;r)\right\rangle }_{s}+{\mathrm{Cov}}_{s}({\left\{\phi \right\}}_{l},\;{\left\{w\right\}}_{l})\\ & \equiv S_{\mathrm{local}}(r)+S_{\mathrm{interlocal}}(r). \end{array}$$

Hence, *S*_local_ is the average of the LSD over all local environments (defined by their midpoints ***m*** and scale *r*), where each environment is weighted by its local density and its local mean fitness:

4.8 $$S_{\mathrm{local}}(r)={\left\langle {\left\{w\right\}}_{l}S_{l}(m;r)\right\rangle }_{s}.$$

It captures the total effect of selection *within* local environments. On the other hand, *S*_interlocal_(*r*) is the spatial covariance (defined in terms of spatial means) of the local mean phenotype and local mean fitness. This term captures the selection *among* environments.

## Data Availability

Simulation codes and scripts to generate and analyse the data are provided as electronic supplementary material [78].
